# Supplementary material for: Rethinking the residual approach: Leveraging machine learning to operationalize cognitive resilience in Alzheimer’s disease
Source: medRxiv. 2024 Aug 20:2024.08.19.24312256. Preprint. [Version 1] doi: 10.1101/2024.08.19.24312256 (PMC11370494; doi:10.1101/2024.08.19.24312256)
Supplement: Supplementary file 1 [file media-1.pdf]

Supplementary Material: Rethinking the residual approach: Leveraging machine learning to operationalize cognitive resilience in Alzheimer's disease

Colin Birkenbihl*[1], Madison Cuppels[1], Rory T. Boyle[2], Hannah M. Klinger[1], Oliver Langford[3], Gillian T. Coughlan[1], Michael J. Properzi[1], Jasmeer Chhatwal[1,4], Julie T. Price[5], Aaron P. Schultz[1,6], Dorene M. Rentz[4], Rebecca E. Amariglio[4], Keith A. Johnson[5], Rebecca F. Gottesman[7], Shubhabrata Mukherjee[8], Paul Maruff[9,10], Yen Ying Lim[9], Colin L. Masters[10], Alexa Beiser[11,12], Susan M. Resnick[13], Timothy M. Hughes[14,15], Samantha Burnham[16], Ilke Tunali[16], Susan Landau[17], Ann D. Cohen[18], Sterling C. Johnson[19,20], Tobey J. Betthauser[19,20], Sudha Seshadri[11,21], Samuel N. Lockhart[14], Sid E. O'Bryant[22], Prashanthi Vemuri[23], Reisa A. Sperling[1,4], Timothy J. Hohman[24,25,26], Michael C. Donohue[3], Rachel F. Buckley[1,4,27]

1. Department of Neurology, Massachusetts General Hospital, Harvard Medical School, Boston, MA 02114, USA.
2. Penn Frontotemporal Degeneration Center, Department of Neurology, Perelman School of Medicine, University of Pennsylvania.
3. Alzheimer Therapeutic Research Institute, University of Southern California, San Diego, USA.
4. Department of Neurology, Center for Alzheimer Research and Treatment, Brigham and Women's Hospital, Harvard Medical School, Boston, MA 02115, USA.
5. Department of Radiology, Massachusetts General Hospital, Harvard Medical School, Boston, MA 02114, USA.
6. Athinoula A. Martinos Center for Biomedical Imaging, Department of Radiology, Massachusetts General Hospital, Charlestown, MA 02129, USA.
7. National Institute of Neurological Disorders and Stroke, Bethesda, MD, USA.
8. Division of General Internal Medicine, Department of Medicine, University of Washington, Seattle, USA.
9. Turner Institute for Brain and Mental Health, School of Psychological Sciences, Monash University, Clayton, Victoria, Australia
10. Florey Institute and the University of Melbourne, Parkville, Victoria, Australia
11. Department of Neurology, Chobanian and Avedisian School of Medicine, Boston University School of Medicine, Boston, MA, USA
12. Department of Biostatistics, Boston University School of Public Health, Boston, USA
13. Laboratory of Behavioral Neuroscience, National Institute on Aging, Baltimore, MD, USA
14. Department of Internal Medicine, Wake Forest School of Medicine, Winston-Salem, NC, USA
15. Alzheimer's Disease Research Center, Wake Forest School of Medicine, Winston-Salem, NC, USA
16. Eli Lilly and Company, Indianapolis, USA
17. Neuroscience Department, University of California, Berkeley, Berkeley, CA, USA
18. Department of Psychiatry, School of Medicine, University of Pittsburgh, 3811 O'Hara Street, Pittsburgh, PA, 15213, USA
19. Department of Medicine, University of Wisconsin-Madison, Madison, Wisconsin, USA
20. Wisconsin's Alzheimer's Disease Research Center, Madison, Wisconsin, USA
21. Glenn Biggs Institute for Alzheimer's & Neurodegenerative Diseases, University of Texas Health Science Center at San Antonio, San Antonio, TX, USA.
22. Institute for Translational Research Department of Pharmacology and Neuroscience, University of North Texas Health Science Center, Fort Worth, TX, USA.
23. Department of Radiology, Mayo Clinic, Rochester, MN 55905, USA.
24. Vanderbilt Memory and Alzheimer's Center, Vanderbilt University Medical Center, Nashville, TN, USA
25. Department of Neurology, Vanderbilt University Medical Center, Nashville, TN, USA
26. Vanderbilt Genetics Institute, Vanderbilt University School of Medicine, Nashville, TN, USA
27. Melbourne School of Psychological Sciences, University of Melbourne, Melbourne, VIC, Australia

Simulation non-linear data

Below, we present the non-linear transformation used to simulate a non-linear relationship between predictors, the expectation component, and the cognitive resilience (CR) component of the observed outcome. We used the function as implemented in scikit-learn[1] and presented in Friedman et al.[2]:

$$y(x) = 10 * \sin(\pi * x_1 * x_2) + 20 * (x_2 - 0.5)^2 + 10 * x_3 + 5 * x_4 + noise * N(0,1)$$

Hyperparameter tuning

We tuned the following hyperparameters in the employed xgboost models using Optuna, a package for Bayesian hyperparameter optimization[3] with the following distributions to sample them from:

- gamma: FloatDistribution(1, 8)
- learning_rate: FloatDistribution(1e-7, 1, log=True)
- max_depth: IntDistribution(3,8)
- subsample: FloatDistribution(0.7, 1, 0.1)

Schematic comparing CR estimates across residual approaches

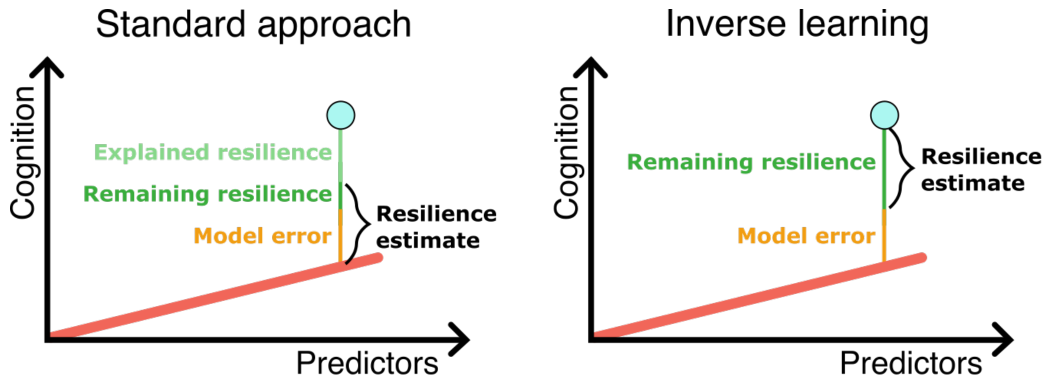

Figure S1: Difference between the CR estimates achieved by the standard approach and the proposed inverse learning approach. The standard approach will 'learn away' parts of the resilience component in the observed cognition due to either direct inclusion of associated predictors or correlation. Further, the linear regression will not perfectly predict the observed cognition and thus introduces model error. The residual achieved by the standard approach will thus contain an unknown proportion of error and have lost a proportion of the sought-after information (resilience). The inverse learning approach will not learn any of the predictor-resilience associations and the model error can be quantified and reduced using the 'error correction' model, thus leading to more reliable estimates of cognitive resilience.

1. Pedregosa, F. *et al.* Scikit-learn: Machine Learning in Python. *J. Mach. Learn. Res.* **12**, 2825–2830 (2011).
2. Friedman, J. H. Multivariate Adaptive Regression Splines.
3. Akiba, T., Sano, S., Yanase, T., Ohta, T. & Koyama, M. Optuna: A Next-generation Hyperparameter Optimization Framework. *ArXiv190710902 Cs Stat* (2019).
